# Reply to Sethuraman et al. Comment on “Utsumi et al. Differences in Pathophysiology and Treatment Efficacy Based on Heterogeneous Out-of-Hospital Cardiac Arrest. *Medicina* 2024, *60*, 510”

**DOI:** 10.3390/medicina60081289

**Published:** 2024-08-09

**Authors:** Shu Utsumi, Mitsuaki Nishikimi, Shinichiro Ohshimo, Nobuaki Shime

**Affiliations:** Department of Emergency and Critical Care Medicine, Graduate School of Biomedical and Health Sciences, Hiroshima University, Hiroshima 734-8551, Japan; asap903@hiroshima-u.ac.jp (S.U.); ohshimos@hiroshima-u.ac.jp (S.O.); shime@koto.kpu-m.ac.jp (N.S.)

We thank the authors of the Letter to the Editor for their astute points raised in reference to our original study [1], “Comment on Utsumi et al. Differences in Pathophysiology and Treatment Efficacy Based on Heterogeneous Out-of-Hospital Cardiac Arrest”. 

First, Sethuraman et al. raised concerns about the cutoff values of NSE for neurological outcomes in Kim et al.’s [2] study. This is because the median was higher in the non-shockable group than in the shockable group, whereas the cutoff for NSE with a false-positive rate of less than 1% was higher in the shockable group than in the non-shockable group. As Sethuraman et al. pointed out, this is an unusual situation, but it is not impossible, as shown in Figure 1. We have shown that with such a small sample of patients with positive test results, results similar to those as in Kim et al.’s study can be obtained. Thus, we cannot conclude that Kim et al. misinterpreted the cutoff value, as the authors stated. We would like to revise the discussion of Kim et al.’s study to the following: “Kim et al. reported that the cutoff values of NSE differed depending on the initial waveform”.

Regarding the second point made by Sethuraman et al., we did indeed incorrectly cite a reference by Kim et al. (reference no. 24 in the text) [2], which should have been Perkins et al. (reference no. 25 in the text) [3]. We apologize for this oversight and will correct this in our article. We appreciate the authors’ suggestions, which have helped us refine our report.

## Figures and Tables

**Figure 1:**
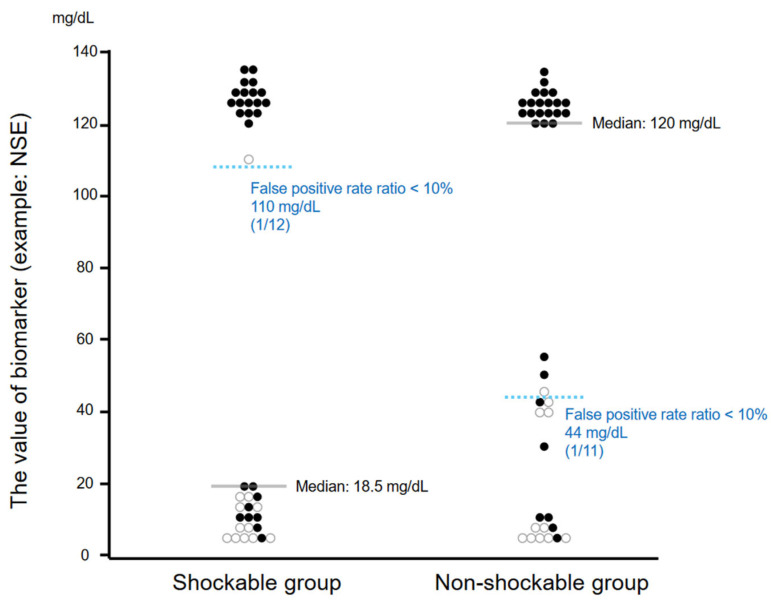
Example of median and cutoff values reversed; Figure 1 shows that a small sample of patients with positive test results can lead to events similar to those seen in Kim et al.’s [2] study. Figure 1 shows the possibility that a reversal situation can occur, with median values lower in the shockable group but cutoff values for NSE with false-positive rates of less than 1% higher in the shockable group. NSE; neuron-specific enolase. It should be noted that Figure 1 is a figure we created and does not contain references from the literature nor uses actual cases.
